# NicheDeSig: niche-aware deconvolution and adaptive signature analysis for spatial transcriptomics

**DOI:** 10.1093/bioinformatics/btag578

**Published:** 2026-07-30

**Authors:** Wen Xue, Juncheng Zhang, Tianyi Chen, Wenjun Shen, Jinjin Ma, Yong Xu, Hau-San Wong, Si Wu

**Affiliations:** School of Computer Science and Engineering, South China University of Technology, Guangzhou, Guangdong 510006, P.R. China; School of Future Technology, South China University of Technology, Guangzhou, Guangdong 510006, P.R. China; Department of Computer Science, City University of Hong Kong, Kowloon 999077, Hong Kong; Department of Bioinformatics, Shantou University Medical College, Shantou, Guangdong 515063, P.R. China; The Institute of Future Health, South China University of Technology, Guangzhou 511442, P.R. China; School of Computer Science and Engineering, South China University of Technology, Guangzhou, Guangdong 510006, P.R. China; Department of Computer Science, City University of Hong Kong, Kowloon 999077, Hong Kong; School of Computer Science and Engineering, South China University of Technology, Guangzhou, Guangdong 510006, P.R. China

## Abstract

**Motivation:**

For spot-based spatial transcriptomics (ST), accurate cell-type deconvolution is essential for downstream analysis since each spot captures mixtures of multiple cell types. Meanwhile, spatial niches define distinct micro-environmental contexts, also salient for biological interpretation. However, existing deconvolution methods usually rely on fixed reference signatures or mapping single cells onto ST spots, without incorporating niche priors or modeling niche-dependent shifts. Consequently, existing methods remain focused on spot-level proportion estimation, with limited ability to support functional analysis of niche-associated molecular programs.

**Results:**

We present NicheDeSig for niche-aware deconvolution. NicheDeSig models each cell type through adaptive signatures, enabling spot deconvolution under context-dependent signatures and supporting niche-aware analysis of cell-state variation across spatial micro-environments. Our method achieves strong deconvolution performance across the simulated benchmark datasets and improves spatial fidelity in the simulated colon dataset. The learned signatures recover laminar and white-matter-associated programs in the human dorsolateral prefrontal cortex (DLPFC), domain-stratified tumor microenvironment patterns in breast cancer (BRCA), and region-associated signatures in pancreatic ductal adenocarcinoma sample A (PDAC-A) and colorectal liver metastasis analyses.

**Availability and implementation:**

Source code and the archived code snapshot are available at https://github.com/Davidcoach/NicheDeSig and https://doi.org/10.5281/zenodo.20685597.

## 1 Introduction

Spatial transcriptomics (ST) (Ståhl *et al.* 2016) enables gene expression profiling while preserving spatial context, making it a powerful tool for studying tissue architecture and micro-environmental heterogeneity. Following the initial ST platforms, numerous sequencing-based methods have been developed to improve spatial resolution and expand the application of spatial omics, including Slide-seqV2 (Stickels *et al.* 2021) and seqFISH+ (Eng *et al.* 2019). These ST technologies facilitate detailed studies of tissue organization in diverse settings such as brain development, organogenesis, and cancer (Chen *et al.* 2025a, 2026). In sequencing-based ST platforms, each spot captures transcripts from multiple cells, resulting in expression profiles that are mixtures of heterogeneous cellular states. Meanwhile, biologically meaningful interpretation requires not only estimating spot composition but also understanding how cell-type programs vary across distinct spatial niches. Consequently, accurate cell-type deconvolution and the modeling of niche-related programs have become foundational steps for downstream ST analysis.

To achieve precise cell-type deconvolution, the reference-based deconvolution methods typically transfer cell-type information from annotated single-cell RNA sequencing (scRNA-seq) data to spatial spots. Traditional reference-based methods like Stereoscope (Andersson *et al.* 2020) and RCTD (Cable *et al.* 2022) model each spot as a mixture of reference-defined cell states (Elosua-Bayes *et al.* 2021), mainly focused on estimating spot-level cell-type proportions. To further improve deconvolution with spatial priors and learned representations, Tangram (Biancalani *et al.* 2021), cell2location (Kleshchevnikov *et al.* 2022), and DestVI (Lopez *et al.* 2022) incorporate spatial priors with deep representation learning for fine-grained cellular mapping. Concurrently, high-resolution assignment based methods such as CytoSPACE (Vahid *et al.* 2023) and others (Zha *et al.* 2025) refine reference-based deconvolution from spot-level composition toward cell-level mapping or alignment, whereas abundance-modeling methods (Chu *et al.* 2022, Chen *et al.* 2025b) couple cell-type abundance estimation with the modeling of cell-type-specific expression variation for more general deconvolution. Recent advances have also broadened ST analysis through masked representation learning, as in SpaMask (Min *et al.* 2025), cross-batch integration, as in SpaBatch (Niu *et al.* 2025), multimodal spatial expression prediction, as in mclSTExp (Min *et al.* 2024), and domain-adversarial deconvolution, as in SpaDAMA (Huang *et al.* 2025). When paired single-cell references are unavailable, non-reference-based methods attempt to recover latent cellular structure directly from spatial data through weakly supervised and unsupervised representation learning (Chen *et al.* 2024, Lin *et al.* 2024, Liu *et al.* 2024, Wei *et al.* 2025). Ultimately, existing deconvolution methods primarily focus on estimating cell-type proportions or improving spatial assignment but do not model niche-dependent shifts in cell-type programs, which limits their utility for functional analysis across different spatial micro-environments.

To characterize spatial niches and their biological associations, early graph-based methods such as STAGATE (Dong and Zhang 2022) integrate gene expression profiles with spatial proximity graphs to establish a foundation for spatial domain identification. Subsequent approaches like GraphST (Long *et al.* 2023) use graph neural networks to learn spatially informed spot representations, enabling a more accurate delineation of tissue structural boundaries. More recently, clustering-based methods including CellCharter (Varrone *et al.* 2024), NicheCompass (Birk *et al.* 2025), and scNiche (Qian *et al.* 2025) incorporate multiple data views to identify multicellular niches and characterize context-dependent molecular programs across distinct anatomical regions. Despite these advances, current analytical pipelines predominantly treat cell-type deconvolution and niche analysis as isolated tasks. Deconvolution methods typically assume static reference signatures to estimate spot-level compositions, while niche identification algorithms operate on fixed clustered embeddings without incorporating adaptive expression modeling from the upstream mixture inference. The isolation between deconvolution and niche analysis makes cross-domain results more sensitive to upstream deconvolution errors and fixed reference assumptions, which may reduce robustness when the same cell type undergoes context-dependent state changes across niches.

In this work, we present NicheDeSig, a niche-aware deconvolution framework that integrates spatial transcriptomics deconvolution with adaptive signature learning. NicheDeSig represents each cell-type signature as a shared global component plus a niche-specific residual, preserving cell-type identity while allowing context-dependent transcriptional shifts. It supports both annotated-niche analysis, such as cortical layers in the human dorsolateral prefrontal cortex (DLPFC), and an unannotated global adaptive-signature mode. We evaluate NicheDeSig on simulated datasets (Li *et al.* 2023), human DLPFC slices, 10x Visium breast cancer (BRCA) data, GSE111672 (Moncada *et al.* 2020) primary pancreatic ductal adenocarcinoma sample A (PDAC-A) data, and GSE225857 (Wang *et al.* 2023) colorectal liver metastasis. Across these analyses, NicheDeSig improves controlled deconvolution benchmarks, reconstructs more faithful simulated-colon spatial organization, and yields interpretable adaptive signatures for tumor-microenvironment program analysis. These results position NicheDeSig as a route from spot composition to cell-type identity and candidate niche-associated molecular programs.

## 2 Materials and methods

We consider spatial transcriptomics (ST) data $X^{st}={\left\{x_{n}^{st}\right\}}_{n=1}^{N}$ with *N* spatial spots and an external scRNA-seq reference $X^{sc}={\left\{x_{m}^{sc}\right\}}_{m=1}^{M}$ with *M* single cells. Each spatial profile $x_{n}^{st}$ and each single-cell profile $x_{m}^{sc}$ lie in $R^{G}$, where *G* denotes the number of genes. Let *C* denote the number of cell types, and let each single cell $x_{m}^{sc}$ be associated with a cell-type label y∈{1,…,C}. For each spatial spot, the goal of deconvolution is to estimate a cell-type proportion vector $p\in R^{C}$, where $p_{i}\geq 0$ for all *i* and $\sum_{i=1}^{C}p_{i}=1$. We define a niche as a local spatial micro-environment characterized by neighborhood composition, tissue architecture, and anatomical position. When niche annotations are available for the ST data, each spot is assigned a niche label k∈{1,…,K}, where *K* denotes the number of niches. NicheDeSig is designed to recover both spot-level cell-type composition and niche-dependent shifts in cell-type programs.

### 2.1 Overview

**Figure 1:**
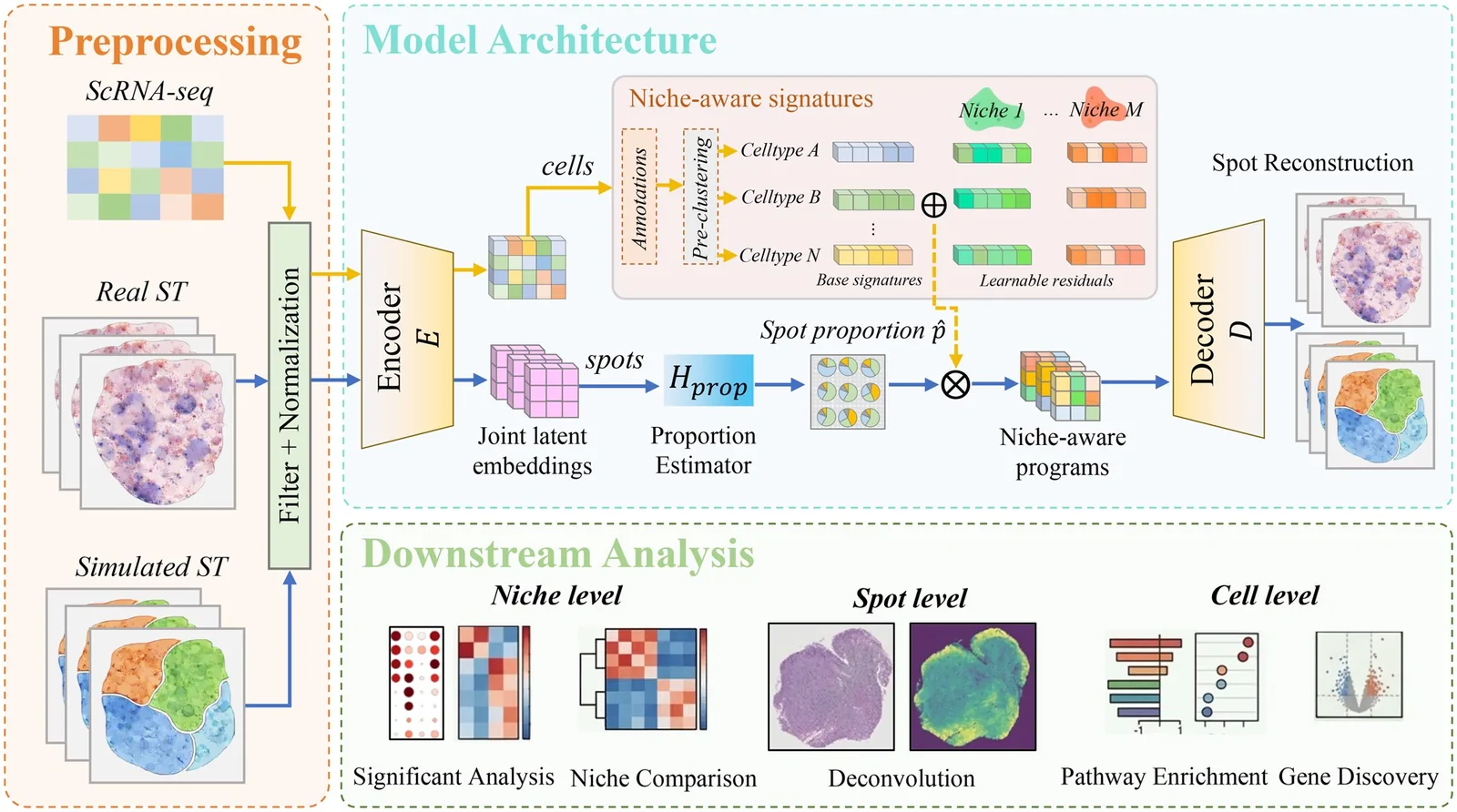
Schematic overview of the NicheDeSig framework. **Left**: Input data include scRNA-seq, real spatial transcriptomics (ST), and simulated ST. **Right Top**: Training pipeline of NicheDeSig. All preprocessed data are projected into a shared latent space for proportion prediction and adaptive signature learning with niche-specific residual signatures. **Right Bottom**: Application of the trained model to real ST data. NicheDeSig supports spot-level deconvolution and downstream analyses at the slice, niche, and gene levels, including cross-slice niche comparison, marker comparison, pathway analysis, and association analysis.

NicheDeSig is a niche-aware deconvolution framework for spatial transcriptomics that estimates spot-level cell-type composition while learning adaptive cell-type signatures under distinct spatial micro-environments. As illustrated in Fig. 1, the framework consists of four main components: an expression encoder E(·), a decoder D(·), a proportion prediction head $H_{\text{prop}}$, and a learnable signature matrix *S* that links predicted cell-type proportions to latent representations. Let *d* denote the latent dimensionality. Given an input expression profile $x\in R^{G}$, the encoder maps *x* to a latent representation:


$$z=E(x),\;z\in R^{d}.$$

For pseudo-ST or real ST spots, the proportion prediction head outputs a cell-type composition vector $\hat{p}$:


(1) $$\hat{p}=H_{\text{prop}}\left(E(x)\right),\;\hat{p}\in R^{C},\;{\hat{p}}_{i}\geq 0,\;\sum_{i=1}^{C}{\hat{p}}_{i}=1,$$

where ${\hat{p}}_{i}$ denotes the *i*th entry of $\hat{p}$. NicheDeSig combines the predicted composition vector $\hat{p}$ with a learnable signature matrix *S* to construct a latent representation for each spot:


(2) $$\hat{z}=\hat{p}\cdot S,\;\hat{x}=D(\hat{z}),$$

where *S* denotes the signature matrix used for reconstruction. In the annotated setting, *S* is instantiated by the niche-adaptive signature matrix $S^{k}$ corresponding to niche *k*, whereas in the unannotated setting it reduces to the global base-signature matrix $S^{\mathrm{base}}$. Under this formulation, the latent representation of each spot is reconstructed as a weighted combination of cell-type signatures, where the weights are given by the predicted cell-type proportions and the signature matrix is determined by the niche setting. In this way, NicheDeSig links spot-level deconvolution with niche-aware signature modeling, enabling the framework to capture both cell-type composition and niche-dependent variation in cell-type programs.

### 2.2 Adaptive signature learning for niche-aware deconvolution

We introduce an adaptive signature formulation in the latent space, allowing the same cell type to preserve a stable core identity while adapting to different spatial micro-environments. For each cell type *i*, the base signature $S_{i}^{\mathrm{base}}$ is initialized by the latent center of scRNA-seq cells annotated as cell type *i*. In annotated-niche analyses, $\Delta S_{i}^{k}$ is initialized with small random values and learned from real ST spots from niche *k*; in unannotated analyses, no niche-specific residual is introduced. The niche-adaptive signature of cell type *i* in niche *k* is defined as:


(3) $$S_{i}^{k}=S_{i}^{\mathrm{base}}+\Delta S_{i}^{k},$$

where $S_{i}^{\mathrm{base}}$ denotes the shared latent prototype of cell type *i* and $\Delta S_{i}^{k}$ captures its niche-dependent variation in niche *k*. Collecting the signatures of all cell types yields the global base-signature matrix $S^{\mathrm{base}}=[S_{1}^{\mathrm{base}};\ldots ;S_{C}^{\mathrm{base}}]\in R^{C\times d}$. For niche *k*, the corresponding niche-adaptive signature matrix is $S^{k}=[S_{1}^{k};\ldots ;S_{C}^{k}]\in R^{C\times d}$. Here, $S_{i}^{\mathrm{base}}$ represents the shared latent prototype of cell type *i*, whereas $\Delta S_{i}^{k}$ captures its niche-dependent deviation in niche *k*. When niche labels are unavailable, NicheDeSig reduces to a global adaptive-signature mode, in which no niche-specific residual is introduced and the reconstruction uses the shared base-signature matrix $S^{\mathrm{base}}$. The reconstructed latent representation is then constrained to match the encoder output as follows:


(4) $$L_{\mathrm{sig}}=E_{x^{st}∼X^{st}}\left[{\left|\left|\hat{z}-E(x^{st})\right|\right|}_{2}^{2}\right].$$

This constraint requires deconvolution predictions to explain each spot through an explicit mixture of biologically interpretable latent signatures, enabling downstream analysis of niche-specific cell states and gene programs.

### 2.3 Pseudo-ST simulation and supervised deconvolution

To provide supervised signals for deconvolution, we simulate pseudo-ST spots from annotated scRNA-seq cells. Following the pseudo-spot strategy used in Spoint (Xu *et al.* 2023), we sample both the number of cells and the number of participating cell types from truncated Poisson distributions, then aggregate selected cells according to the sampled composition. This yields pseudo-spots with explicit proportion labels and heterogeneous mixture complexity.

For each pseudo spot, we first sample a target composition vector *p*, then randomly sample cells, aggregate their profiles, and constrain the total expression to match the library size of real ST data:


(5) $$x^{\mathrm{fake}}=\sum_{j=1}^{L}w_{j}x_{j}^{sc},\;s.t.\;|x^{\mathrm{fake}}|*1=\ell ,\;\ell ∼P*lib^{st},$$

where *L* denotes the number of sampled cells, $\overset{sc}{\underset{j}{x}}$ denotes the sampled scRNA-seq profiles, $w_{j}$ denotes the aggregation weight of cell *j*, and ℓ is sampled from the empirical library-size distribution of real ST spots. This formulation constrains pseudo-ST simulation by both cell-type composition and library size. To avoid narrow synthetic mixtures, we over-generate candidate pseudo-spots and retain the top 10% candidates ranked by their similarity to real ST spots in library size and detected-gene count.

The pseudo-ST profiles are designed to provide controlled training examples with known cell-type proportions. The resulting pseudo-ST dataset provides composition labels and is used to supervise the proportion predictor, and $\hat{p}$ denotes the predicted proportions:


(6) $$\begin{array}{cc} \hat{p}=H_{\text{prop}}\left(E(x^{\mathrm{fake}})\right), & \\ L_{\mathrm{prop}}=E_{(x,p)∼X^{\mathrm{fake}}}\left[||H_{\text{prop}}(E(x^{\mathrm{fake}}))-p|{|}_{1}\right]. & \end{array}$$

### 2.4 Model optimization

NicheDeSig is trained with a three-stage optimization strategy that explicitly separates shared representation learning, global deconvolution learning, and niche-specific signature refinement. Before describing the staged training procedure, we summarize the main loss functions used in the framework. The reconstruction loss $L_{\mathrm{rec}}$ is used to preserve the major biological structure of the input data in the latent space by requiring the decoder to recover the original expression profile from the latent embedding:


(7) $$L_{\mathrm{rec}}=E_{x∼X^{sc}\cup X^{st}}[|\left|x-D(E(x))\right|{|}_{2}^{2}].$$

In addition, NicheDeSig incorporates the pseudo-ST proportion supervision loss $L_{\mathrm{prop}}$ in Equation (6) and the signature reconstruction loss $L_{\mathrm{sig}}$ in Equation (4) to constrain spot-level deconvolution and adaptive signature learning, respectively. Building on these loss terms, NicheDeSig is optimized in three progressive stages.

In the first stage, we pretrain the encoder *E* and decoder *D* using scRNA-seq cells and real ST spots. The goal of this stage is to learn a stable shared latent space that preserves major biological variation and provides a reliable initialization for subsequent deconvolution and signature learning. This autoencoder pretraining stage is performed for 60 epochs using AdamW with a learning rate of $1\times 10^{-3}$, a batch size of 512, and masked reconstruction in which 75% of input genes are randomly masked at each iteration. During this stage, only the encoder–decoder reconstruction pathway is updated, while the proportion prediction head and signature parameters are kept inactive:


(8) $$\underset{E,D}{\min}\;L_{\mathrm{rec}}.$$

In the second stage, the pretrained encoder and decoder are kept fixed, and the proportion prediction head $H_{\text{prop}}$ together with the global base-signature matrix $S^{\mathrm{base}}$ are optimized using pseudo-ST and real ST inputs. This stage is trained for 20 epochs with a main learning rate of $2\times 10^{-5}$. The goal of this stage is to learn spot-level deconvolution under pseudo-ST supervision while establishing global base signatures as adaptive cell-type prototypes. The corresponding optimization objective is:


(9) $$\begin{array}{cc} \underset{\;H_{\text{prop}},\;S^{\mathrm{base}}}{\min} & L_{\mathrm{prop}}+L_{\mathrm{sig}}. \end{array}$$

where $L_{\mathrm{sig}}$ is computed using the global base-signature matrix $S^{\mathrm{base}}$ at this stage.

In the third stage, when niche labels are available, NicheDeSig performs niche-specific residual refinement for 50 epochs with a learning rate of $1.5\times 10^{-5}$. The encoder, decoder, and global base-signature matrix $S^{\mathrm{base}}$ are kept fixed to preserve the shared latent space and global cell-type backbone. The proportion prediction head remains a single shared $H_{\text{prop}}$ across all niches and continues to receive global pseudo-ST proportion supervision, because pseudo-ST spots are not divided by niche. Only the residual signatures $\Delta \overset{k}{\underset{i}{S}}$ are niche indexed and are refined using real ST spots assigned to niche *k*. This stage isolates niche-dependent transcriptional variation around each cell-type backbone and enables niche-aware signature analysis of within-cell-type molecular programs. For each niche *k*, the optimization objective is:


(10) $$\underset{H_{\text{prop}},{\left\{\Delta S_{i}^{k}\right\}}_{i=1,\ldots ,C}}{\min}L_{\mathrm{prop}}+L_{\mathrm{sig}}^{\left(k\right)},$$

where $H_{\text{prop}}$ is shared across niches, and $\overset{\left(k\right)}{\underset{\mathrm{sig}}{L}}$ is computed using the niche-adaptive signature matrix $S^{k}$. When niche labels are unavailable, this stage is skipped and NicheDeSig operates in the global adaptive-signature mode using $S^{\mathrm{base}}$. Overall, this three-stage training strategy allows NicheDeSig to first establish a stable latent representation, then achieve quantitatively reliable deconvolution, and finally refine interpretable niche-aware signatures for downstream biological analysis.

### 2.5 Downstream analysis

After training, NicheDeSig supports downstream analysis by projecting the learned latent signatures back to the original gene space. Let $\overset{\mathrm{base}}{\underset{i}{S}}$ denote the base signature of cell type *i* and let $\Delta \overset{k}{\underset{i}{S}}$ denote the corresponding niche-specific residual for niche *k*. The gene-space base signature of cell type *i* is defined as:


(11) $$\mu_{i}^{\mathrm{base}}=D_{\phi }(S_{i}^{\mathrm{base}})\in R^{G},$$

and the niche-adaptive gene-space signature $\overset{\left(k\right)}{\underset{i}{\mu }}$ together with the corresponding gene-level deviation $\Delta \overset{\left(k\right)}{\underset{i}{\mu }}$ are given by:


(12) $$\Delta \mu_{i}^{k}=\mu_{i}^{k}-\mu_{i}^{\mathrm{base}},$$

where $\Delta \overset{\left(k\right)}{\underset{i}{\mu }}$ quantifies how the molecular program of cell type *i* shifts from its shared base state to niche *k*. In the unannotated global adaptive-signature mode, no niche-specific residual is introduced and the analysis reduces to the shared gene-space signature $\overset{\mathrm{base}}{\underset{i}{\mu }}$.

### 2.6 Reproducibility and implementation

NicheDeSig was implemented in Python using a deep-learning framework and trained on an NVIDIA GeForce RTX 3090 GPU. As described above, the same preprocessing pipeline, including normalization, log-transformation, and shared feature selection, was applied to both scRNA-seq and spatial transcriptomics data. Pseudo-ST generation, model training, and downstream analyses were performed using fixed manuscript-specific scripts and configuration files provided in the project repository.

All experiments were conducted with fixed random seeds to improve reproducibility. Final results for the benchmark evaluations were reported on the corresponding held-out test sets defined for each benchmark. For baseline comparisons, official implementations or author-released code were used whenever available.

## 3 Results

To evaluate NicheDeSig, we benchmark deconvolution performance across 32 simulated datasets (Li *et al.* 2023) and a simulated colon dataset (Shen *et al.* 2026), then test biological interpretability on DLPFC, BRCA, GSE111672 PDAC-A (Moncada *et al.* 2020), and GSE225857 colorectal liver metastasis (Wang *et al.* 2023). DLPFC evaluates annotated-niche laminar programs, BRCA evaluates unannotated global adaptive signatures, PDAC-A tests label-free hematoxylin and eosin (H&E)-region recovery and region-associated signatures, and GSE225857 examines annotation-guided tumor microenvironment composition and signature-derived gene programs in colorectal liver metastasis. Deconvolution performance is evaluated using Pearson correlation coefficient (PCC), Jensen–Shannon divergence (JS), root mean square error (RMSE), and structural similarity index measure (SSIM); higher PCC/SSIM and lower JS/RMSE indicate better performance. Table 1 summarizes the dataset sources, tissue contexts, spot counts, and paired scRNA-seq references.

**Table 1:** Datasets used in the experimental analyses.^a^

Dataset	Tissue/context	Spatial data	scRNA-seq reference
32 simulated benchmark datasets (Li *et al.* 2023)	Multi-tissue simulated ST benchmark from Li et al.	32 datasets; 1000 spots per dataset	943–10 000 cells per dataset; 11 725–30 355 genes
Simulated colon pseudo-ST (Shen *et al.* 2026)	Colon pseudo-ST simulation for spatial-fidelity evaluation and ablation analysis	4 pseudo-ST slices; 4519/5357/5141/5279 spots; 20 296 spots in total	5981 cells; 1076–1360 genes
Human DLPFC (Maynard *et al.* 2021)	Human dorsolateral prefrontal cortex from Maynard et al.	8 Visium sections, 151 669–151 676; 29 648 in-tissue spots	78 886 single-nucleus cells; 30 062 genes
10x Visium BRCA (Wu *et al.* 2021)	Human breast cancer from 10x Genomics Human Breast Cancer Visium dataset	3798 in-tissue Visium spots	45 647 cells; 5000 selected genes
GSE111672 PDAC-A (Moncada *et al.* 2020)	Pancreatic ductal adenocarcinoma from GEO GSE111672	426 spots with region labels	1926 inDrop cells; 14 121 aligned genes
GSE225857 colorectal liver metastasis (CRLM) (Wang *et al.* 2023)	Colorectal cancer liver metastasis and primary tumor from GEO GSE225857	6 visium sections; 22 260 spots with niche labels	1800 sampled cells; 988 genes

a Counts refer to the processed analysis-ready inputs used in this study.

### 3.1 Quantitative performance across benchmark datasets

We first ask whether NicheDeSig can serve as a reliable deconvolution model, since downstream signature interpretation is only meaningful if the underlying proportion estimates are reliable. We benchmark NicheDeSig against representative deconvolution baselines, including probabilistic methods such as DestVI (Lopez *et al.* 2022), cell2location (Kleshchevnikov *et al.* 2022), and Stereoscope (Andersson *et al.* 2020), regression-based methods such as Tangram (Biancalani *et al.* 2021) and RCTD (Cable *et al.* 2022), and recent spatial deconvolution methods including Spoint (Xu *et al.* 2023) and SpaDAMA (Huang *et al.* 2025). The evaluation is conducted across 32 simulated benchmark datasets and an independent simulated colon dataset with ground-truth cell-type proportions.

**Figure 2:**
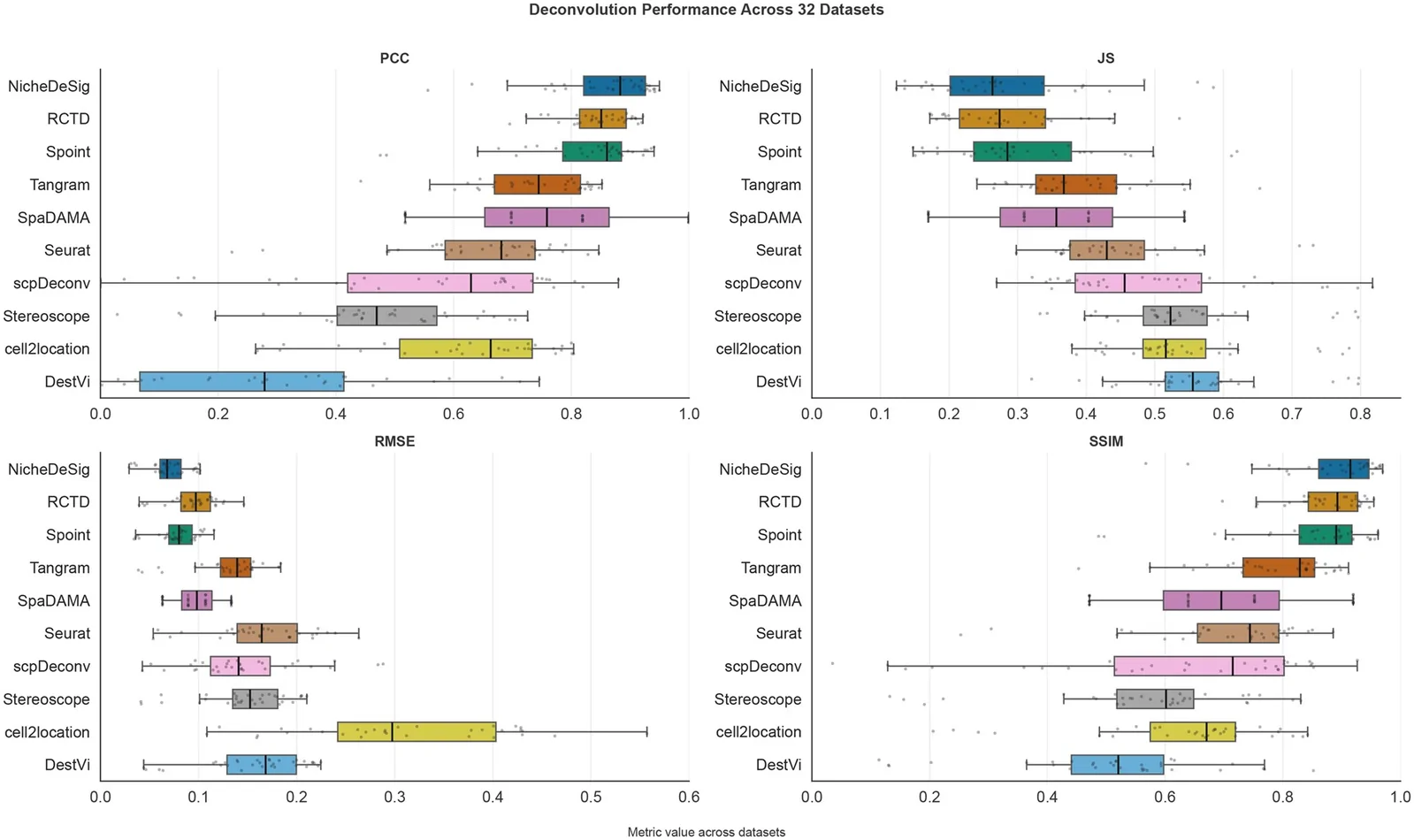
Quantitative comparison of competing methods across 32 simulated benchmark datasets. Boxplots report Pearson correlation coefficient (PCC↑), Jensen–Shannon divergence (JS↓), root mean square error (RMSE↓), and structural similarity index measure (SSIM↑). Higher PCC and SSIM and lower JS and RMSE indicate better performance.

As shown in Table 2, NicheDeSig achieves the best mean performance across the 32 simulated benchmark datasets, with PCC/JS/RMSE/SSIM values of 0.8557/0.2813/0.0706/0.8834. Compared with the best competing method for each metric, NicheDeSig improves PCC by 1.1% relative to Spoint, reduces JS by 3.6% relative to Spoint, lowers RMSE by 9.7% relative to RCTD, and increases SSIM by 0.7% relative to Spoint. The boxplots in Fig. 2 further show favorable medians and compact interquartile ranges, especially for JS and RMSE, indicating robust performance across heterogeneous simulated settings.

**Table 2:** Performance summary on the main benchmark datasets.^a^

Method	Simulated benchmarks				Simulated Colon			
	PCC ↑	JS ↓	RMSE ↓	SSIM ↑	PCC ↑	JS ↓	RMSE ↓	SSIM ↑
Tangram	0.7352 ± 0.0974	0.3926 ± 0.0963	0.1310 ± 0.0369	0.7837 ± 0.1047	0.5346 ± 0.0211	0.5938 ± 0.0418	0.1673 ± 0.0136	0.5498 ± 0.0187
Stereoscope	0.4627 ± 0.1707	0.5369 ± 0.1180	0.1466 ± 0.0465	0.5629 ± 0.1782	0.5648 ± 0.0237	0.5547 ± 0.0217	0.1420 ± 0.0047	0.5830 ± 0.0303
cell2location	0.6083 ± 0.1752	0.5346 ± 0.1065	0.3129 ± 0.1100	0.6265 ± 0.1676	0.6048 ± 0.0347	0.6843 ± 0.0526	0.1621 ± 0.0025	0.4915 ± 0.0246
DestVI	0.2749 ± 0.2421	0.5619 ± 0.1097	0.1581 ± 0.0515	0.5091 ± 0.1777	0.7732 ± 0.0588	0.3809 ± 0.0330	0.0643 ± 0.0081	0.7777 ± 0.0571
RCTD	0.8186 ± 0.1164	0.3116 ± 0.1218	0.0782 ± 0.0205	0.8474 ± 0.1183	0.8300 ± 0.0205	0.3671 ± 0.0323	0.0699 ± 0.0062	0.8338 ± 0.0208
Seurat	0.6456 ± 0.1402	0.4502 ± 0.0977	0.1655 ± 0.0517	0.6959 ± 0.1440	0.8175 ± 0.0385	0.3727 ± 0.0279	0.0802 ± 0.0018	0.8240 ± 0.0386
scpDeconv	0.5479 ± 0.2382	0.4949 ± 0.1467	0.1474 ± 0.0596	0.6244 ± 0.2391	0.6410 ± 0.0395	0.5269 ± 0.0231	0.1261 ± 0.0040	0.6568 ± 0.0393
Spoint	0.8464 ± 0.0953	0.2918 ± 0.1173	0.0940 ± 0.0256	0.8771 ± 0.0957	0.8589 ± 0.0296	0.3549 ± 0.0265	0.0660 ± 0.0029	0.8626 ± 0.0295
SpaDAMA	0.7585 ± 0.1783	0.3564 ± 0.1383	0.0983 ± 0.0260	0.6955 ± 0.1660	0.8350 ± 0.0235	0.3681 ± 0.0176	0.0782 ± 0.0061	0.7858 ± 0.0291
**NicheDeSig**	**0.8557** ± **0.0584**	**0.2813** ± **0.0918**	**0.0706** ± **0.0203**	**0.8834** ± **0.0626**	**0.8870** ± **0.0178**	**0.3138** ± **0.0203**	**0.0554** ± **0.0008**	**0.8897** ± **0.0204**

a Pearson correlation coefficient (PCC), Jensen–Shannon divergence (JS), root mean square error (RMSE), and structural similarity index measure (SSIM) are reported as mean ± standard deviation. The best result in each metric is shown in bold and the second-best result is underlined.

The same trend is observed on the simulated colon dataset. NicheDeSig obtains the best PCC/JS/RMSE/SSIM values of 0.8870/0.3138/0.0554/0.8897. Relative to the best competing method for each metric, NicheDeSig improves PCC by 3.3% over Spoint, reduces JS by 11.6% over Spoint, lowers RMSE by 13.8% over DestVI, and increases SSIM by 3.1% over Spoint. Compared with SpaDAMA, NicheDeSig also improves PCC, JS, RMSE, and SSIM by 6.2%, 14.8%, 29.2%, and 13.2%, respectively. Together, these results show that NicheDeSig provides a quantitatively reliable deconvolution backbone across simulated benchmarks with known ground truth, supporting its subsequent use for niche-aware signature interpretation.

### 3.2 Spatial fidelity in the simulated Colon dataset

**Figure 3:**
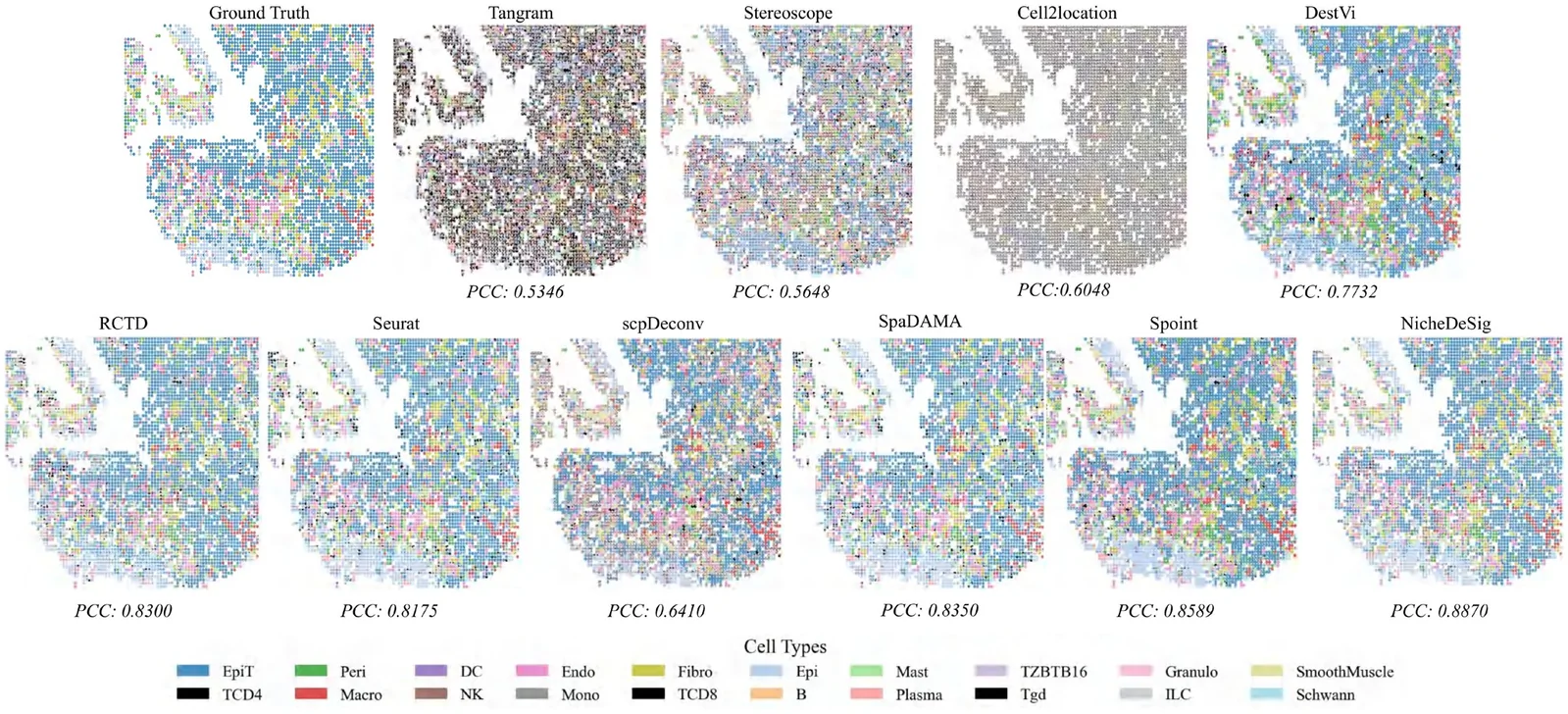
Qualitative comparison of deconvolution results on the simulated colon dataset. The upper-left panel shows the simulated ground truth, and the color legend below indicates the annotated cell types. Black pixels denote single-cell states that are present in the scRNA-seq reference but absent from the simulated ST ground truth. A better deconvolution result should therefore recover the spatial organization of the simulated ground truth while reducing transfer of these reference-only states. Compared with competing methods, NicheDeSig provides a controlled qualitative view of spatial organization and reference-only state transfer, complementing the quantitative summaries in Table 2.

We also compare the qualitative deconvolution results on the simulated colon dataset, where **black** denotes reference-only cell states that appear in the scRNA-seq reference but not in the simulated ST ground truth. As shown in Fig. 3, NicheDeSig preserves the global tissue layout, reduces transfer of reference-only states, and maintains compartment continuity and boundary geometry more consistently than the displayed baselines. This visual pattern is consistent with Table 2, where NicheDeSig attains the best PCC/SSIM and lowest JS/RMSE on the same colon simulation. We interpret the visualization results as spatial fidelity with respect to simulated ground truth.

### 3.3 Niche-associated molecular programs in DLPFC

**Figure 4:**
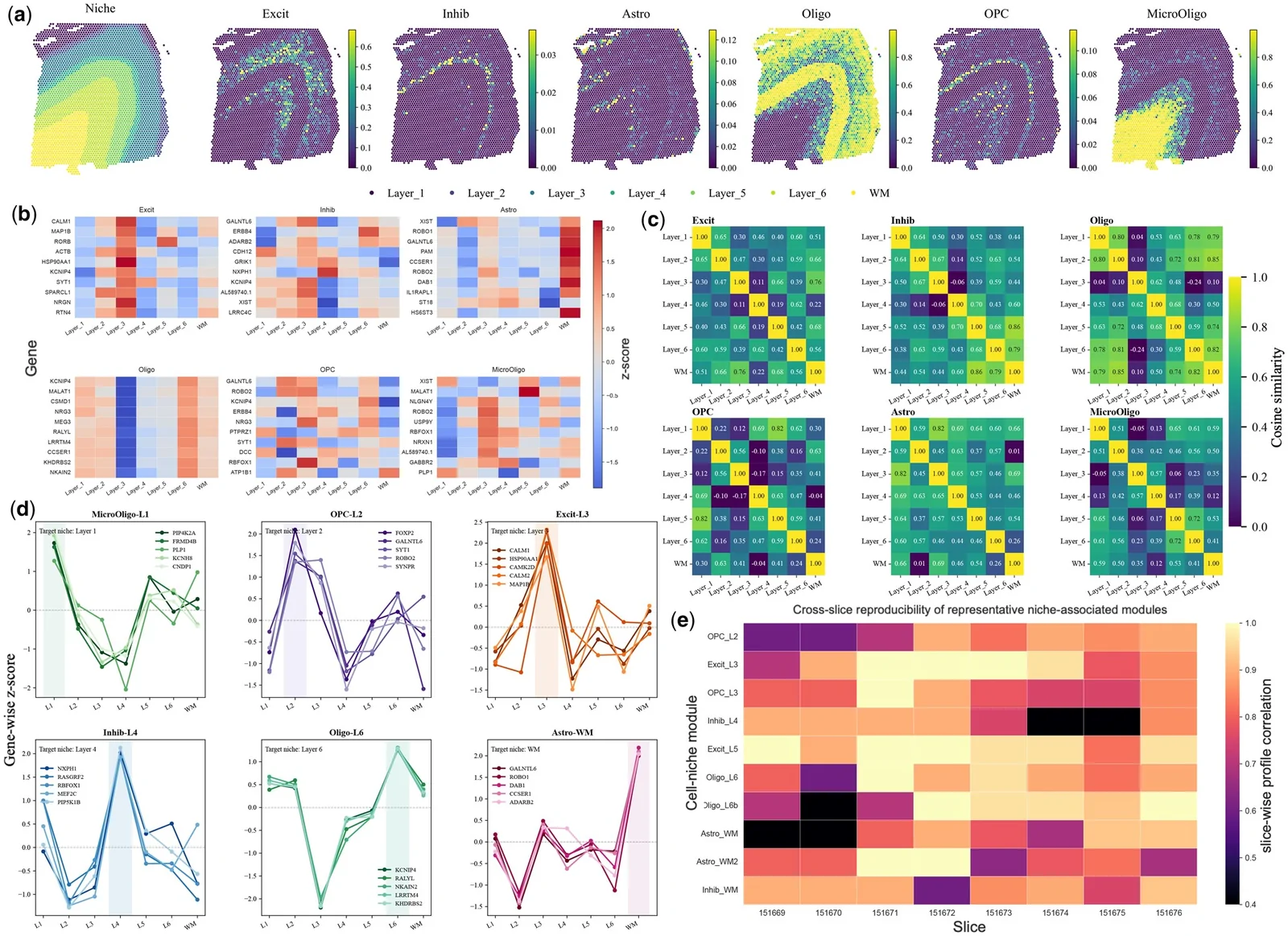
NicheDeSig resolves niche-conditioned molecular programs and cross-slice reproducibility in human DLPFC. (a) Niche annotation and signature maps for major cell types in slice 151673. (b) Gene-by-niche heatmaps showing layer- and white-matter-dependent signature shifts within each cell type. (c) Within-cell-type niche-similarity matrices showing structured laminar relationships. (d) Representative multi-gene trajectories highlighting coordinated niche-associated modules. (e) Cross-slice reproducibility of representative niche modules across eight DLPFC sections (151669–151676). These panels show that NicheDeSig captures within-cell-type variation, preserves laminar organization, and recovers reproducible niche-associated programs across DLPFC sections.

To evaluate NicheDeSig in a structured tissue context, we applied it to human DLPFC (Maynard *et al.* 2021), using cortical layer annotations as niche labels. As shown in Fig. 4a–c, joint training across eight slices recovers layer- and white-matter-associated signatures for major lineages, including excitatory neurons, inhibitory neurons, oligodendrocytes, astrocytes, and progenitor-related populations. Within each cell type, the learned signatures vary systematically across niches rather than collapsing to a single prototype. The niche-similarity matrices further preserve the laminar organization of the tissue, with adjacent layers showing greater similarity than distant layers or white matter.

Gene-level analysis shows that the recovered niche variation is coordinated across multi-gene programs. In Fig. 4d, programs such as Excit-L3, Inhib-L4, Oligo-L6, OPC-L2, Astro-WM, and MicroOligo-L1 show niche-aligned peaks or troughs. Figure 4(e) further shows that representative programs maintain high slice-wise profile correlations while preserving layer-associated differences.

These results suggest that NicheDeSig signatures capture reproducible laminar and white-matter-associated molecular programs across cell types, layers, and tissue sections, providing interpretable evidence for structured niche-associated variation in DLPFC.

### 3.4 Domain-specific tumor microenvironment programs in BRCA

**Figure 5:**
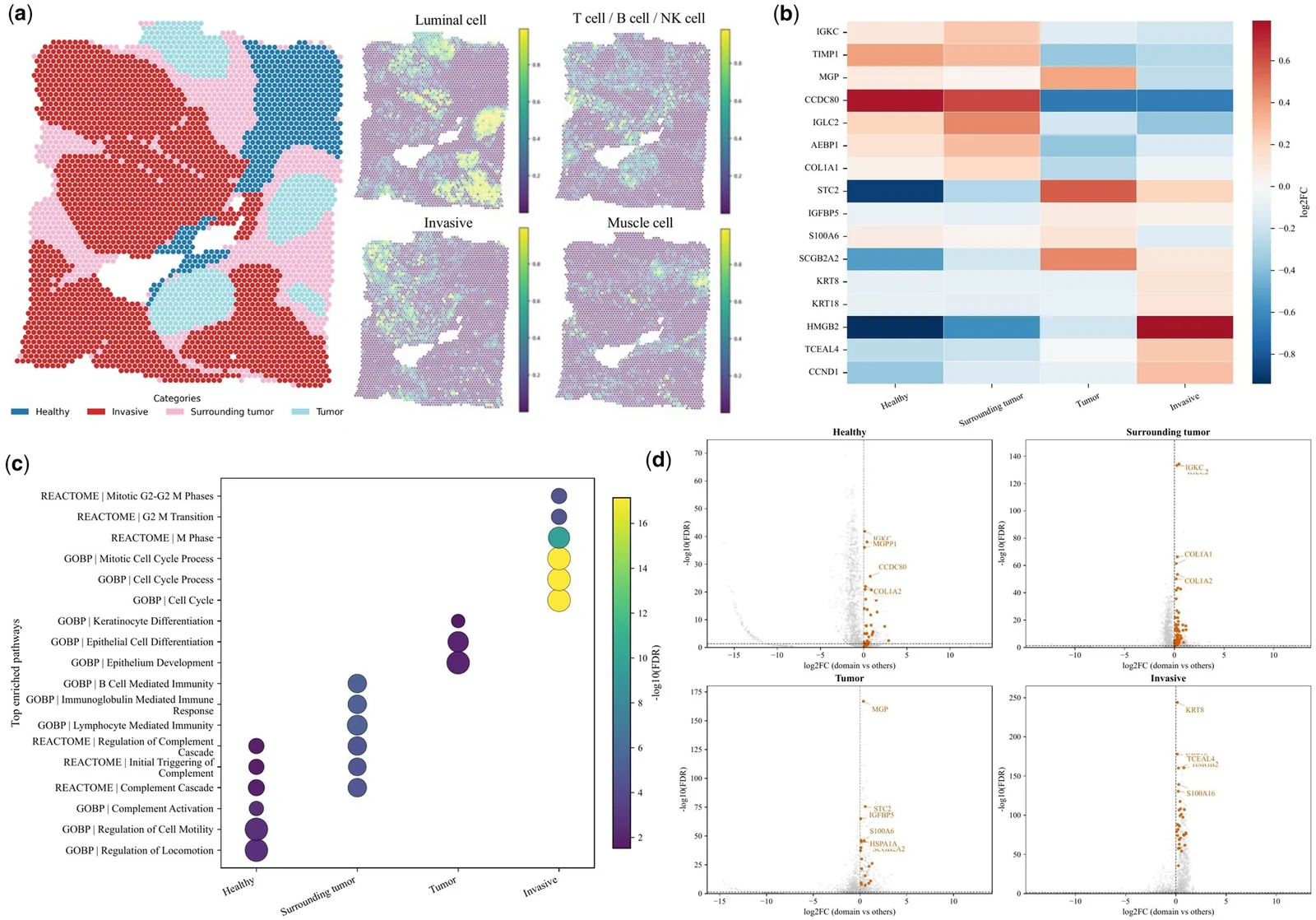
NicheDeSig reveals domain-specific cellular programs and gene-level patterns in human breast cancer. (a) Domain annotation and representative signature maps for Luminal cell, T-, B-, and natural killer (NK)-cell, Invasive, and Muscle cell programs. (b) Representative domain-associated genes across Healthy, Surrounding tumor, Tumor, and Invasive regions. (c) Enriched pathways derived from domain-associated signatures; bubble size denotes overlap gene count and color denotes $-{\;\text{log}\;}_{10}$ of the false discovery rate (FDR). (d) Domain-wise volcano plots with representative key genes highlighted. These panels support domain-associated interpretation by connecting spatial domains, decoded signature programs, pathway enrichment, and gene-level differences.

To assess unannotated settings, we applied NicheDeSig in global adaptive-signature mode to human BRCA sections. As shown in Fig. 5a, NicheDeSig recovers coherent domain-associated patterns across Healthy, Surrounding tumor, Tumor, and Invasive regions. Tumor-associated regions are dominated by luminal and invasive epithelial signals, surrounding regions show stronger stromal and immune structure, and healthy regions retain non-malignant background characteristics. Figure 5b shows that these domains differ in associated molecular programs: Healthy regions retain vascular and homeostatic markers, Surrounding tumor is enriched for extracellular-matrix and stromal-remodeling genes, and Tumor/Invasive regions display epithelial and proliferative transcriptional patterns.

Pathway and differential-gene analyses provide additional context. In Fig. 5c, Healthy region is associated with vascular and endogenous-response programs, Surrounding tumor with extracellular-matrix organization and collagen remodeling, and Invasive region with cell-cycle and mitosis pathways. Figure 5d shows the same progression at the gene level, including proliferative markers such as *BIRC5*, *CCNB1*, *CENPF*, *TOP2A*, and *UBE2C*.

### 3.5 Region-associated deconvolution and molecular programs in PDAC-A

**Figure 6:**
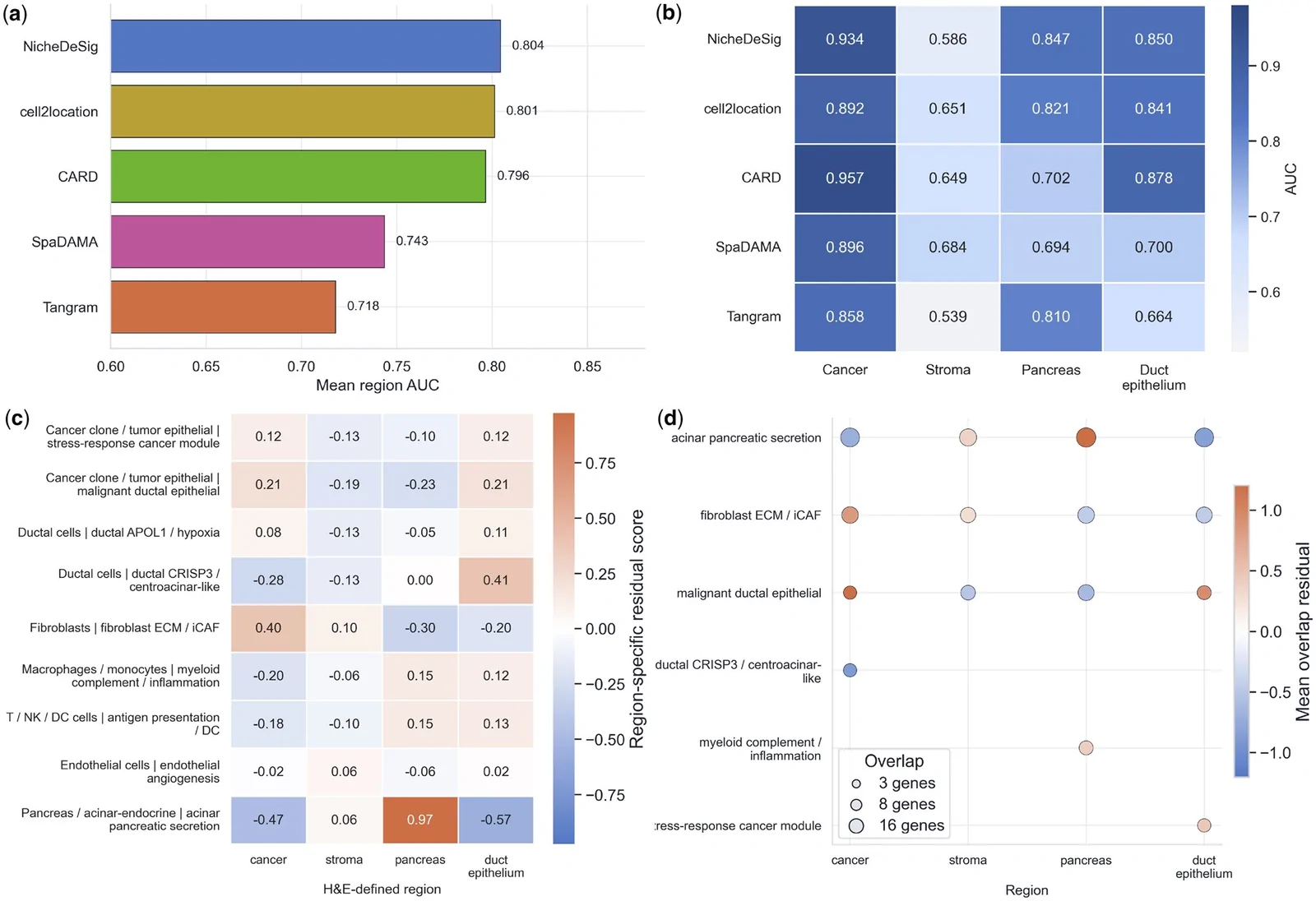
(a) Mean one-versus-rest area under the receiver operating characteristic curve (AUC) for H&E-derived anatomical regions in GSE111672 PDAC-A. Region labels were withheld during model training and prediction and used only for post hoc validation; NicheDeSig achieves the highest mean AUC among the displayed methods. (b) Region-specific AUCs for cancer, stroma, pancreas, and duct epithelium, showing stronger recovery of cancer, pancreas, and duct epithelium and a remaining challenge in stromal recovery. (c) Annotated-niche signature heatmap using H&E-derived region annotations as niche labels. The learned signature matrices highlight region-associated programs, including malignant ductal/cancer, CRISP3/centroacinar-like ductal, pancreatic/acinar secretion, endothelial/stromal angiogenesis, and partial fibroblast extracellular-matrix (ECM) and inflammatory cancer-associated fibroblast (iCAF) signals. (d) Gene-set enrichment dot plot summarizing signature-derived region-associated programs. Point size denotes overlap size, and color denotes the mean direction of the corresponding signature-derived genes.

We further evaluated NicheDeSig on the PDAC-A dataset to assess whether accurate deconvolution can be linked to adaptive signature interpretation in an independent disease context. The analysis used 1926 inDrop scRNA-seq cells and 426 ST spots with 14 121 aligned genes. For label-free spatial deconvolution, H&E-derived anatomical region labels were withheld during training and inference, and were used only after prediction to compute one-versus-rest areas under the receiver operating characteristic curve (AUCs) for cancer, stroma, pancreas, and duct epithelium. As shown in Figure 6a and b, NicheDeSig achieved the highest mean region AUC among the displayed methods (0.804), outperforming cell2location (0.801), CARD (0.796), SpaDAMA (0.743), and Tangram (0.718). Region-specific results showed strong recovery of cancer, pancreas, and duct epithelium regions, whereas stroma remained the most challenging compartment. When endothelial cells and red blood cells were included in an expanded stromal mapping, NicheDeSig reached a higher mean AUC of 0.823.

After the label-free deconvolution benchmark, we further used the H&E-derived region annotations as niche labels to run NicheDeSig in its annotated-niche mode. This analysis was conducted separately from the AUC benchmark, where the same annotations were withheld during training and prediction and used only for post hoc validation. Under the annotated-niche setting, NicheDeSig learned region-aware signature matrices that captured spatially organized molecular programs. As shown in Fig. 6c and d, the signature heatmap linked malignant ductal epithelial programs to cancer and ductal regions, CRISP3/centroacinar-like programs to duct epithelium, and acinar secretion programs to pancreas. Additional region-associated signals, including endothelial/stromal angiogenesis and partial fibroblast extracellular-matrix (ECM) and inflammatory cancer-associated fibroblast (iCAF) programs, were also observed. The enrichment dot plot summarized the overlap size, statistical significance, and direction of the signature-derived gene sets, supporting candidate region-associated molecular programs. Together, the PDAC-A analysis shows that NicheDeSig can perform label-free deconvolution while using H&E-defined niche annotations in the annotated mode to derive interpretable region-associated signatures.

### 3.6 Tumor microenvironment programs in colorectal liver metastasis

**Figure 7:**
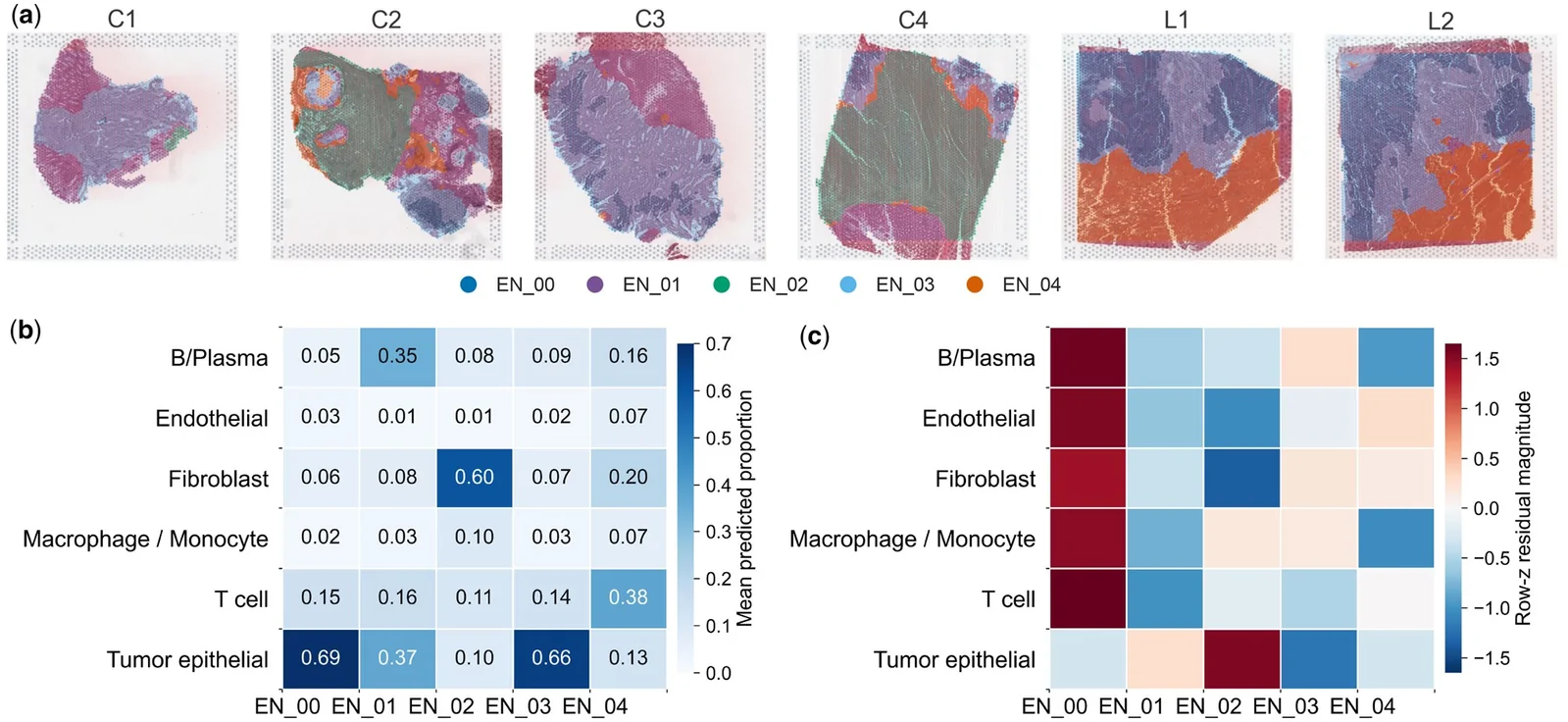
GSE225857 colorectal liver-metastasis analysis using spot-level annotations. (a) Annotation overlays on H&E images across six sections (C1–C4 and L1–L2), showing spatially structured niche patterns. (b) Heatmap of mean predicted cell-type composition across annotated niches, highlighting tumor-epithelial-enriched EN_00/EN_03, fibroblast-enriched EN_02, B/plasma–tumor mixed EN_01, and immune/stromal mixed EN_04. (c) Row-normalized heatmap of signature-derived gene programs decoded from the learned signature matrices, revealing cell-type-specific molecular patterns associated with tumor epithelial, fibroblast, immune, and stromal contexts across annotated niches.

We further evaluated NicheDeSig on the GSE225857 colorectal liver-metastasis Visium dataset. In this analysis, the provided spot-level annotations were used as spatial contexts for downstream signature analysis. Figure 7a shows annotation overlays across six H&E sections. The composition heatmap in Fig. 7b identifies several dominant cellular contexts: EN_00 and EN_03 are enriched for tumor epithelial signals, EN_02 is fibroblast enriched, EN_01 contains mixed B/plasma and tumor epithelial signals, and EN_04 shows an immune/stromal mixed pattern.

We next decode the learned signature matrices into gene space to examine whether NicheDeSig captures niche-associated molecular programs beyond mean cell-type composition. As shown in Fig. 7c, the gene-level signatures reveal cell-type-specific variation across annotated niches, highlighting molecular programs associated with tumor epithelial, fibroblast, immune, and stromal contexts. These results provide a hypothesis-generating colorectal liver-metastasis example in which NicheDeSig links spatial annotations, deconvolution outputs, and signature-derived gene programs.

### 3.7 Pseudo-ST realism analysis

**Figure 8:**
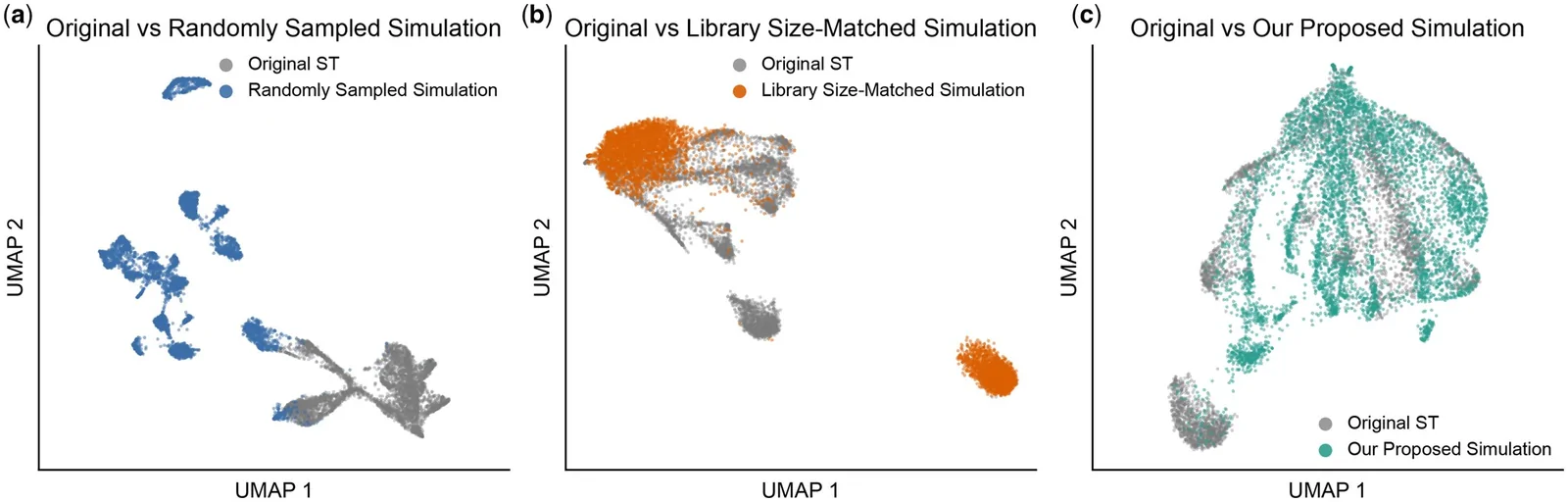
UMAP visualization of real ST and synthetic pseudo-ST profiles from three simulation strategies: (a) random aggregation, (b) library-size matching, and (c) the proposed pseudo-ST simulation. Random aggregation remains separated from the real-spot manifold, library-size matching corrects the intended scale mismatch but remains displaced, and the proposed pseudo-ST profiles show stronger qualitative overlap. Quantitative QC summaries are reported in Table 3; pseudo-ST is interpreted as controlled supervision with known proportions, not as a complete simulator of ST measurement noise or spatial dependence.

Since NicheDeSig uses pseudo-ST profiles as supervised training targets for deconvolution, we evaluated whether the simulated profiles provide a suitable scaffold for learning spot-level cell-type composition. We compared three pseudo-ST generation strategies: random scRNA aggregation, library-size-matched simulation, and the proposed pseudo-ST simulation. As shown in Fig. 8, random aggregation remains clearly separated from the real ST manifold, whereas the proposed pseudo-ST profiles show stronger qualitative overlap with real ST spots. Table 3 further shows that the proposed strategy effectively removes the library-size gap and improves the mean QC KS statistic relative to simple library-size matching. The detected-gene and zero-fraction KS values remain relatively high, indicating that pseudo-ST profiles do not fully reproduce all technical properties of real ST measurements. We therefore use pseudo-ST as controlled and diverse supervision with known cell-type proportions, rather than as a complete simulator of spatial measurement noise or spatial dependence.

**Table 3:** Pseudo-ST calibration summary.^a^

Synthetic strategy	Library-size KS ↓	Detected-gene KS ↓	Zero-fraction KS ↓	Mean QC KS ↓
Random scRNA aggregation	0.708	0.557	0.510	0.592
Library-size matching	0.013	0.605	0.605	0.408
Proposed pseudo-ST	0.001	0.513	0.553	0.356

a KS statistics measure QC agreement between synthetic and real ST profiles.

### 3.8 Ablation study of NicheDeSig variants

To assess the contribution of different NicheDeSig components, we evaluated complementary model variants on the simulated colon dataset as shown in Table 4. All variants were trained to convergence using the same settings and evaluated using the same input data, pseudo-ST sampling strategy, checkpoint selection procedure, and evaluation metrics. The full-model row uses the same simulated-colon NicheDeSig values reported in Table 2. The full model achieved PCC/JS/RMSE/SSIM values of 0.8870/0.3138/0.0554/0.8897. Removing $L_{\mathrm{prop}}$ produced the largest PCC decrease, whereas removing Stage 3 caused the largest deterioration in JS, RMSE, and SSIM. This result indicates that explicit pseudo-ST proportion supervision is essential for anchoring spot-level composition estimates. Removing the learnable base signature, Stage 3, $L_{\mathrm{sig}}$, $L_{\mathrm{rec}}$ also reduced performance, supporting the importance of reconstruction-guided signature learning and niche-specific residual refinement.

**Table 4:** Simulated-colon ablation results.^a^

Variant	PCC ↑ (Δ↓)	JS ↑ (Δ↑)	RMSE ↑ (Δ↑)	SSIM ↑ (Δ↓)
Full model	0.887 –	0.314 –	0.055 –	0.890 –
w/o base signature	0.655 (0.232)	0.575 (0.261)	0.121 (0.066)	0.659 (0.231)
w/o $L_{\text{sig}}$	0.694 (0.193)	0.546 (0.232)	0.111 (0.056)	0.699 (0.191)
w/o $L_{\text{rec}}$	0.721 (0.166)	0.519 (0.205)	0.104 (0.049)	0.727 (0.163)
w/o $L_{\text{prop}}$	0.521 (0.366)	0.620 (0.306)	0.146 (0.091)	0.544 (0.346)
w/o Stage 3	0.567 (0.320)	0.662 (0.348)	0.231 (0.176)	0.493 (0.397)

a Values are means across four slices; parentheses denote deterioration relative to the full model.

**Figure 9:**
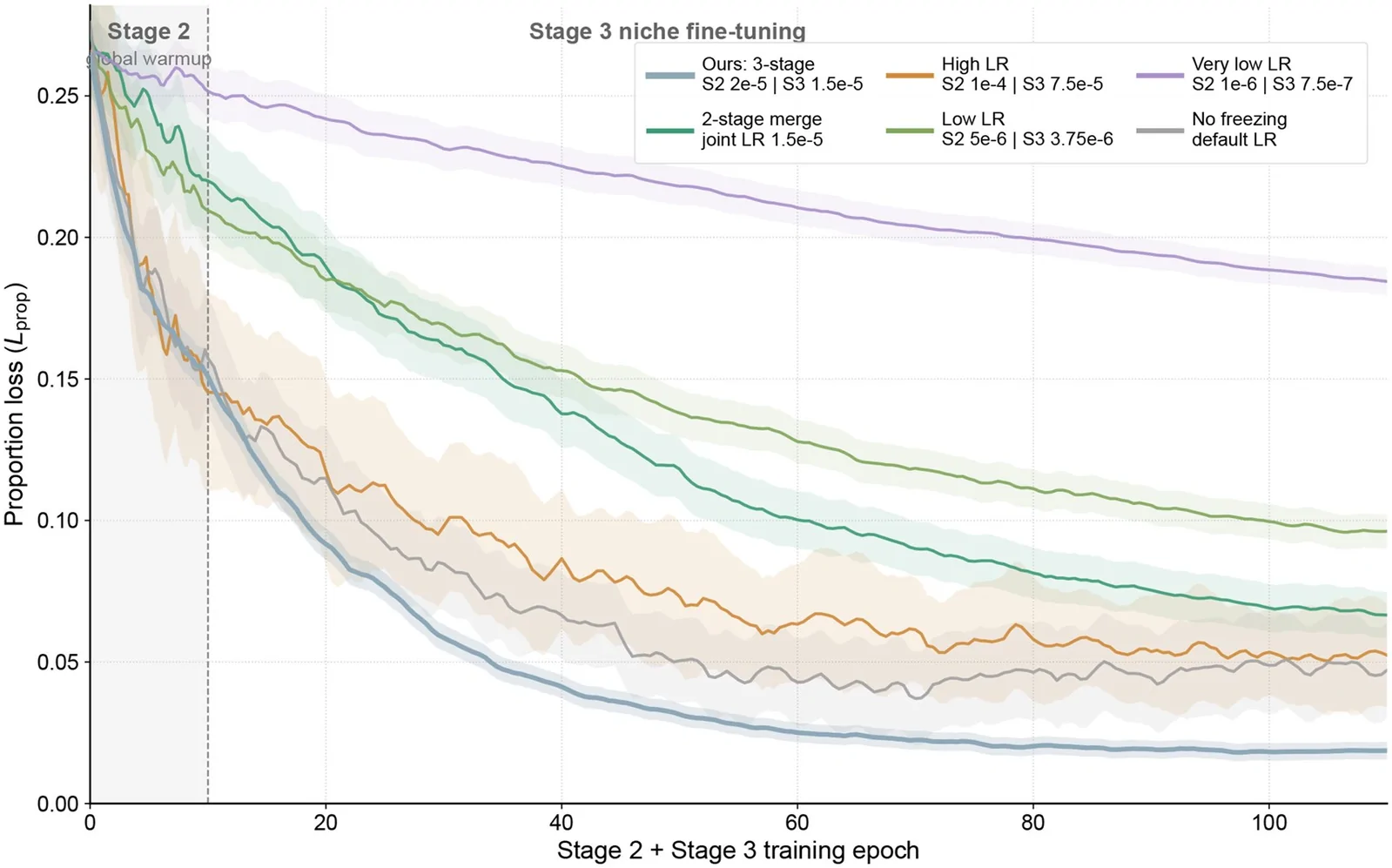
Proportion-loss convergence diagnostics. The default NicheDeSig schedule reaches the lowest terminal proportion loss among the displayed settings and avoids the larger oscillation observed with larger learning rates or with continuous encoder–decoder updating.

We further examined the stability of Stage 2 and Stage 3 optimization under alternative training settings as shown in Table 5 and Fig. 9. The default staged schedule achieved the lowest final proportion loss (0.0186), outperforming merged-stage training, high-learning-rate training, very-low-learning-rate training, and variants without encoder–decoder freezing. These diagnostics are intended to evaluate optimization stability rather than to serve as an additional deconvolution accuracy benchmark.

**Table 5:** Convergence analysis under different training settings.^a^

Setting	Stage-2 LR	Stage-3 LR	Final $L_{\text{prop}}↓$	Late SD↓
NicheDeSig	2e − 5	1.5e − 5	0.0186	0.0031
Merged Stage 2 + 3	Merged	1.5e − 5	0.0679	0.0085
Larger LR	1e − 4	7.5e − 5	0.0522	0.0186
Smaller LR	5e − 6	3.75e − 6	0.0963	0.0062
Very small LR	1e − 6	7.5e − 7	0.1855	0.0051
No Enc./Dec. freezing	2e − 5	1.5e − 5	0.0482	0.0171

a Final $L_{\text{prop}}$ summarizes the convergence level of the proportion-supervision objective, and Late SD reports the late-stage standard deviation of $L_{\text{prop}}$. Lower values indicate better convergence and more stable optimization.

Finally, we profiled computational efficiency on the DLPFC dataset under a matched local RTX 3090 workflow as reported in Table 6. Runtime, peak central processing unit (CPU) resident set size (RSS), and peak graphics processing unit (GPU) memory are reported as mean ± estimated standard deviation across eight DLPFC slices. Together, the ablation results, convergence diagnostics, and resource profiling show that $L_{\mathrm{prop}}$ anchors accurate spot-level deconvolution, whereas the learnable base signatures, $L_{\mathrm{sig}}$, and Stage 3 residual refinement support the proposed base-plus-residual adaptive signature design at a practical computational cost.

**Table 6:** Computational efficiency comparison on the DLPFC dataset.^a^

Method	Runtime	Peak CPU RSS	Peak GPU memory
Tangram	1.00 ± 0.08 min	0.72 ± 0.06 GB	0.11 ± 0.02 GB
SpaDAMA	54.31 ± 6.84 min	7.26 ± 0.58 GB	2.43 ± 0.22 GB
Stereoscope	1.78 ± 0.21 min	1.44 ± 0.11 GB	0.32 ± 0.05 GB
cell2location	14.93 ± 1.76 min	2.56 ± 0.19 GB	0.73 ± 0.07 GB
scpDeconv	23.56 ± 2.88 min	2.66 ± 0.22 GB	1.00 ± 0.09 GB
Spoint	17.52 ± 2.14 min	5.33 ± 0.47 GB	6.74 ± 0.61 GB
DestVI	10.23 ± 1.25 min	1.43 ± 0.10 GB	0.71 ± 0.06 GB
NicheDeSig	3.57 ± 0.42 min	1.64 ± 0.13 GB	3.92 ± 0.35 GB

a Values are reported as mean ± estimated standard deviation across eight slices. Peak central processing unit (CPU) resident set size (RSS) and graphics processing unit (GPU) memory are provided as practical diagnostics of computational resource requirements.

## 4 Discussion

In this study, we presented NicheDeSig, a niche-aware deconvolution framework that integrates spatial transcriptomics deconvolution with adaptive signature learning to connect spot-level mixture inference with niche-oriented biological interpretation. By representing each cell-type program as a shared base signature with niche-dependent adaptation, NicheDeSig separates stable cell-type identity from context-associated molecular variation. This design helps address a key limitation of fixed reference signatures, which may confound abundance changes with niche-dependent transcriptional shifts when the same cell type changes across spatial micro-environments. NicheDeSig combines spot deconvolution and niche-associated latent representation learning with an explicit signature formulation, providing a direct route from quantitative deconvolution to interpretable downstream analysis. The benchmark results should therefore be interpreted at two levels. Across the 32 simulated benchmark datasets, the gains are moderate but consistent, suggesting improved robustness across heterogeneous simulated settings. In the simulated colon dataset, the larger gains and improved agreement with ground truth indicate that adaptive signatures are particularly useful when spatial compartments contain complex mixtures and reference-only states need to be suppressed.

The real-data analyses clarify how NicheDeSig should be interpreted under different spatial settings. In annotated tissues such as DLPFC, predefined cortical layers provide biologically meaningful niche labels, and the learned signatures recover within-cell-type variation aligned with laminar and white-matter organization. The consistency of representative programs across multiple DLPFC sections further supports that these patterns reflect reproducible tissue organization rather than slice-specific noise. In unannotated settings such as BRCA, the global adaptive-signature mode reveals coherent domain-stratified tumor microenvironment patterns, but the interpretation should remain hypothesis-generating because niche labels are not directly observed. The PDAC-A and GSE225857 analyses further extend this calibrated interpretation. In PDAC-A, the region-level deconvolution benchmark is performed in a label-free manner, with H&E-derived anatomical regions withheld during training and prediction and used only for post hoc validation. We then use the H&E-derived region annotations as niche labels in the annotated mode to decode the learned signature matrices into gene space and identify region-associated molecular programs. In GSE225857, the provided spot-level annotations organize predicted cell-type compositions into spatially structured domains, and the decoded signature matrices reveal cell-type-specific gene programs across colorectal liver-metastasis sections. Together, these analyses illustrate how NicheDeSig connects deconvolution outputs with annotation-guided signature interpretation while distinguishing validation labels, spatial annotations, and hypothesis-generating biological programs.

The experiments also help clarify why niche-aware deconvolution is useful beyond descriptive visualization. Accurate deconvolution remains the foundation of ST analysis, but composition estimates alone are often insufficient for explaining how molecular programs vary across spatial micro-environments. By anchoring deconvolution outputs to adaptive and interpretable signatures, NicheDeSig enables niche-aware signature analysis, including cross-slice comparison, gene-level deviation analysis, and pathway-level interpretation. The ablation analyses further support this interpretation: pseudo-ST proportion supervision anchors spot-level composition, whereas the base signatures, signature reconstruction loss, and Stage 3 niche-specific refinement support stable adaptive signature learning. The convergence diagnostics indicate that the staged optimization schedule stabilizes proportion learning and signature refinement, and the computational profiling shows that these components can be trained at practical resource cost. Thus, the contribution of NicheDeSig is not limited to improved proportion estimation. NicheDeSig provides a signature-based representation through which cell-type identity, niche context, and gene-level programs can be analyzed together.

Several limitations should be noted. First, NicheDeSig relies on an external scRNA-seq reference, and discrepancies between dissociated reference profiles and in situ transcriptional states may limit the recovery of niche-specific signals. Second, although pseudo-ST simulation provides supervised training targets, it cannot fully eliminate the domain gap between synthetic and real spatial measurements. The pseudo-ST realism analysis supports its use as controlled supervision with known proportions, but also shows that pseudo-ST profiles do not fully reproduce all technical and spatial properties of real ST measurements. Third, in the annotated setting, the learned residual signatures depend on externally defined niche labels, which may affect both their granularity and biological interpretation. In unannotated or annotation-guided tumor analyses, the resulting programs should be viewed as candidate functional annotations rather than causal or clinical conclusions. Finally, the current framework does not yet incorporate histology or image-derived information into signature learning. Future work could extend NicheDeSig to jointly model multiple slices or samples, improve robustness to reference mismatch, incorporate more realistic pseudo-ST simulation or domain adaptation, and integrate transcriptomic and imaging signals within a niche-aware signature-learning framework. Such extensions may further strengthen the utility of adaptive signatures for functional and hypothesis-generating analysis in larger multi-sample spatial transcriptomics studies.

## 5 Conclusion

We presented NicheDeSig, a niche-aware deconvolution framework that couples spot-level proportion estimation with adaptive signature learning. By representing each cell-type program as a shared base signature plus a niche-specific residual, the method supports both accurate deconvolution and downstream interpretation across annotated, unannotated, and data-driven niche settings. Across simulated benchmarks and the simulated colon dataset, NicheDeSig improves quantitative accuracy and spatial fidelity, while the DLPFC and BRCA case studies show that the learned signatures can support biologically coherent niche- or domain-aware interpretation. The PDAC-A and colorectal liver metastasis analyses further support region-level recovery and adaptive-signature interpretation in tumor microenvironment contexts. Overall, NicheDeSig provides a practical route from spot composition to cell-type identity and candidate niche–cell–gene programs in spatial transcriptomics.

## Data Availability

The data underlying this article are available as follows. The 32 benchmark datasets used for quantitative evaluation follow the public benchmark collection assembled by Li *et al.* (2023). The simulated colon dataset used for qualitative evaluation is a study-specific simulation. The human DLPFC spatial transcriptomics data are publicly available through https://research.libd.org/spatialLIBD/. The paired single-nucleus RNA-seq reference can be accessed through the same resource. The 10x Visium breast cancer spatial dataset is publicly available from the 10x Genomics Human Breast Cancer Whole Transcriptome Analysis dataset. The PDAC-A data are publicly available from GEO under accession GSE111672. The colorectal liver-metastasis Visium and scRNA-seq inputs are publicly available from GEO under accession GSE225857. Processed datasets have been deposited in Zenodo. The data-package DOI is https://doi.org/10.5281/zenodo.20604815.

## Code availability

Source code, manuscript-specific analysis scripts, and processed outputs are available at https://github.com/Davidcoach/NicheDeSig. The archived code snapshot for this revision is available at https://doi.org/10.5281/zenodo.20685597.
